# Effects of *Lactobacillus coryniformis* K8 CECT5711 on the immune response to influenza vaccination and the assessment of common respiratory symptoms in elderly subjects: a randomized controlled trial

**DOI:** 10.1007/s00394-017-1573-1

**Published:** 2017-11-09

**Authors:** Juristo Fonollá, Carlos Gracián, Jose A. Maldonado-Lobón, Carlos Romero, Alicia Bédmar, Juan C. Carrillo, Carmen Martín-Castro, Antonio L. Cabrera, Jose M. García-Curiel, Carlos Rodríguez, Sara Sanbonmatsu, Mercedes Pérez-Ruiz, Jose M. Navarro, Mónica Olivares

**Affiliations:** 1 Biosearch Life, Camino de Purchil 66, 18004 Granada, Spain; 2 Nursing home “Residencia de Mayores Claret”, Granada, Spain; 3 Nursing home “Residencia Entreálamos”, Granada, Spain; 4 Nursing home “Residencia de Mayores San Juan de Dios”, Granada, Spain; 5 Nursing home “Residencia Hermanitas de los Pobres”, Granada, Spain; 6 Nursing home “Residencia Fray Leopoldo”, Granada, Spain; 7 Microbiology Service of Hospital “Virgen de las Nieves”, Institute of Biosanitary Research of Granada, Granada, Spain

**Keywords:** Antiviral immunity, Immunomodulation, Probiotic, Respiratory viruses, Vaccine

## Abstract

**Purpose:**

Elderly people are particularly vulnerable to seasonal influenza. Therefore, vaccination is strongly recommended. However, the vaccine efficacy is lower in the elderly, owing to immunosenescence. The objective of the present study was to evaluate the ability of the probiotic strain *Lactobacillus coryniformis* K8 CECT5711 to enhance the immune response to the influenza vaccine in the elderly and to assess the effects on symptoms related to respiratory infections.

**Methods:**

A randomized, double-blind, placebo-controlled trial was conducted between November 2015 and April 2016. A total of 98 nursing home residents, more than 65 years of age were randomly assigned to receive *L. coryniformis* K8 CECT5711 (3 × 10^9^ CFU/day) or a placebo for 2 weeks before influenza vaccination. The primary outcome was the percentage of seroconversion. The secondary outcomes were the incidence of influenza-like illness (ILI) and respiratory symptoms associated with respiratory infections during the 5-month follow-up period. The serum cytokine and immunoglobulin levels were also evaluated.

**Results:**

The percentage of responders to vaccination was higher in the probiotic group than in the control group (*p* = 0.036). *L. coryniformis* ingestion was associated with a significantly lower incidence of respiratory symptoms commonly associated with respiratory infections (*p* = 0.007) and lower consumption of analgesics (*p* = 0.008).

**Conclusion:**

The administration of *L. coryniformis* K8 CECT5711 to an elderly population increased the immune response against the influenza vaccine and decreased symptoms associated with respiratory infections. Probiotic administration may be a natural and safe strategy to improve the efficacy of vaccines and to protect against common respiratory infections in susceptible populations.

## Introduction

The influenza A and B viruses are important human respiratory pathogens. Influenza occurs globally with an annual attack rate estimated at 5–10% in adults and 20–30% in children [1]. The elderly are particularly vulnerable to seasonal influenza; indeed, approximately 90% of all influenza-related deaths occur among senior citizens [2]. Vaccination remains the most effective public health measure to decrease the effect of seasonal influenza and is strongly recommended for this population [1]. However, the efficacy of the vaccine is lower in the elderly, owing to the immunosenescence characteristic of this population [3, 4]. For this reason, the use of adjuvants has been proposed to enhance the immune response to the influenza vaccine in the elderly. However, the injection of the adjuvant together with the vaccine can increase the incidence of undesirable local or systemic responses [5].

The *Lactobacillus* genus is a common inhabitant of the human gut. Interactions between some representatives of this bacterial genus and the immune system have been demonstrated to enhance the immune response [6]. This activity would be involved in the preventive effect of certain probiotic strains on respiratory infections, as shown in different trials [7–11]. The ability of certain *Lactobacillus* strains to modulate the immune response has also been used to increase the response to vaccines by acting as adjuvants [12, 13]. The use of an oral probiotic administration strategy would avoid the adverse effects associated with the direct injection of chemical adjuvants.

*Lactobacillus coryniformis* K8 CECT5711 (*Lc* K8) is a strain originally isolated from an artisan goat cheese [14]. This strain has been found to have immunomodulatory activity in a previous study in which fermented milk containing *Lc* K8 in combination with the strain *L. gasseri* CECT5711 was administered to healthy adults and enhanced both innate and specific immune responses [15]. Two additional studies performed in children with the same fermented milk containing the combination of *Lactobacillus* strains corroborated the effect on the immune system [16, 17]. Recently, oral administration of *Lc* K8 as a food supplement to healthy adults has been reported to increase specific antibodies against hepatitis A virus after a vaccination protocol [18]. The objective of the present study was to evaluate the ability of Lc K8 to enhance the immune response to the influenza vaccine in the elderly and to assess the effects on respiratory symptoms related to respiratory infections.

## Materials and methods

### Study design

A randomized double-blinded placebo-controlled multicentre trial was performed. The study was started in October 2015 and ended in April 2016.

Volunteers were recruited from five nursing homes in Granada (Spain) at the beginning of the vaccination programme. The inclusion criteria were nursing home residents more than 65 years of age. The exclusion criteria were frequent gastrointestinal diseases; antibiotic treatment during the intervention; allergy to any group of antibiotics, egg proteins or adjuvants; and excipients of the flu vaccine. The study was conducted according to the Declaration of Helsinki, and the protocol was approved by the Regional Ethical Committee (Granada, Spain). Informed consent was obtained from all subjects. The trial was registered in the US Library of Medicine (http://www.clinicaltrial.gov) under number NCT03167593.

The volunteers were randomly assigned to one of two groups according to a randomization scheme generated by a computer programme (SIGESMU^®^). The individuals in the placebo group consumed a capsule containing 300 mg of maltodextrin daily, whereas the individuals in the probiotic group consumed a capsule containing 3 × 10^9^ colony forming units of strain *L. coryniformis* K8 CECT5711 (Lc K8) in a matrix of the same maltodextrin mixture, daily. The probiotic and placebo were provided in identical gelatine capsules packaged in identical plastic containers with a code number that referred to the volunteer code according to the randomization. The capsules were kept at 4 °C to maintain the stability of the viable bacteria concentration in the product. The consumption of any probiotic supplement was restricted from 2 weeks before the beginning of the intervention until the end of the study. During the 2 weeks before flu vaccination, the volunteers received a capsule of probiotic or placebo daily. On day 15 of the study, all volunteers received intramuscular vaccination against the flu. The vaccination was conducted by the medical services of the nursing homes with a vaccine containing inactivated trivalent influenza (A/California/7/22009[H1N1]pdm09, A/HongKong/4801/2014[H3N2], and B/Brisbane/60/2002) for the vaccine campaign of 2015/2016 (Sanofi Pasteur Europe, Lyon, France). All volunteers were vaccinated during the same week (second week of November 2015). After vaccination, the volunteers were followed up until 30 April 2016.

The sample size was estimated according to the effect on the main outcome of the study (seroconversion). On the basis of the expected level, the study was designed to exhibit sufficient power (80%) to detect a difference between groups of 30% in the proportion of seroconverters with a 0.05 significance level (R software version 2.12.2). The number of volunteers necessary was 43 per group, and the total sample size was increased to 100 volunteers to compensate for dropouts.

### Study outcomes

The primary outcome of the study was the percentage of seroconversion. According to the European Centre for Disease Prevention and Control (ECDC), for a population older than 60 years of age, seroconversion corresponds to the proportion of vaccinated individuals achieving a haemagglutination inhibition (HAI) titre > 1:40 or a significant increase in the HAI antibody titre (i.e., at least a four-fold titre increase) [19].

The secondary outcome was the incidence of influenza-like illness (ILI) during the follow-up period. The ILI diagnosis was based on the case definition used by the European Centre for Disease Prevention and Control (as mandated by the European Union for communicable disease reporting, is henceforth abbreviated EU-ECDC) as follows: sudden onset of symptoms with one or more respiratory symptoms (cough, sore throat and/or shortness of breath) plus one or more systemic symptoms (self-reported fever, headache, myalgia and/or malaise) [20].

Other outcomes were the levels of cytokines (IL-10, IL-4, and TNF-alpha), immunoglobulin A (IgA) and immunoglobulin G (IgG).

Gastrointestinal manifestations, such as nausea, vomiting and lack of appetite, and the consumption of analgesics and antibiotics during the follow-up period were also recorded.

All data about the health conditions of the volunteers and the consumption of medical treatments were evaluated and recorded by a medical doctor in the case report form corresponding to each volunteer.

### Collection of blood samples

After an overnight fast lasting at least 10 h, blood samples were collected from the volunteers at the beginning of the intervention (day 0), just before vaccination (day 14), and during follow-up (days 42 and 70), using Vacutainers (S-Monovette, Sarstedt, Germany) containing ethylenediaminetetraacetic acid.

### Haemagglutination inhibition (HAI) test

The reagents for the HAI test were from a World Health Organization Influenza Reagent Kit (Geneva, Switzerland). The influenza antigens contained in the kit are suitable for the serological diagnosis of influenza A(H1), A(H3), and B infections. The HAI tests were performed using standard procedures with chicken red blood cells according to the manual published by the WHO [21].

### Total immunoglobulin and cytokine measurements

The total IgA and IgG concentrations in the plasma were measured in duplicates with enzyme-linked immunosorbent assay (ELISA) quantification kits, according to the manufacturer’s instructions (Bethyl, Montgomery, TX, USA).

The cytokine and interleukin concentrations in the plasma were measured in duplicates with ELISA quantitation kits, according to the manufacturer´s instructions (Affymetrix eBioscience, San Diego, CA, USA).

### Statistical analysis

For seroconversion, the odd ratios (ORs) and 95% confidences intervals (CI) were obtained to assess the effect of treatment. A logistic model was performed with adjustment for age and sex.

The occurrence of illness and symptoms and the consumption of analgesic and antibiotics were described using the incidence ratio (IR) and incidence rate ratio (IRR) with the 95% CI and p value for the IRR. A Poisson regression model was applied to adjust the number of events by sex, age and seroconversion.

For the cytokine and immunoglobulin concentrations, data were obtained at each time point of the study [baseline, 2 weeks (just before vaccination), 6 and 10 weeks]. The descriptive analyses for the responses are shown as the mean and standard error. The 95% CI for the mean and the bivariate statistical test to evaluate differences between groups at each time point were performed using bootstrap methods. Adjusted analysis regression mixed models were applied with a fixed time, group and seroconversion effect. Because regression mixed models require meeting the assumption of normality for the residuals, a log 10 transformation of the responses was performed for IgA and IgG.

A 5% significance level was considered for the statistical tests.

The statistical SPSS version 19 (for the descriptive and bivariate analyses) and R 3.1 (for the modelling data) software were used to perform the analyses.

## Results

**Fig. 1:**
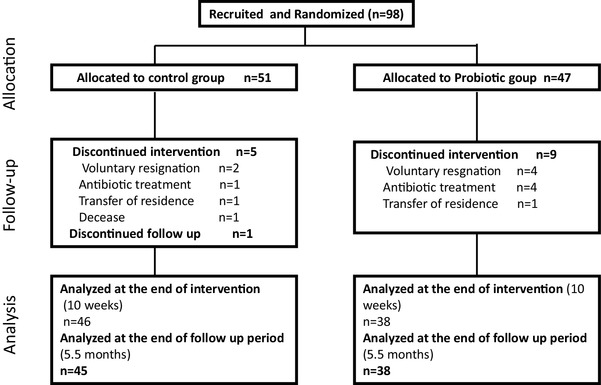
Participant flow diagram for the study

A total of 98 subjects were recruited from 5 elderly residence homes and they were randomly distributed into two groups: the control group and the probiotic group. Before completion of the 10-week intervention period, 6 volunteers in the control group and 9 in the probiotic group discontinued the intervention and dropped out of the study for the reasons detailed in the study flow chart (Fig. 1). Afterwards, one additional subject discontinued the study during the follow-up period in the control group. The reasons for drop out were similar in both groups. The most common reason was voluntary resignation, owing to refusal to have blood drawn. Finally, data from 84 volunteers were included for the analysis of immune responses, and data from 83 volunteers were included for the analysis of the incidence of symptoms of infection.

No significant differences were detected in the baseline characteristics of the volunteers (Table 1). Most of the volunteers, 68%, were older than 80 years, 28% were between 70 and 80 years old and 4% were between 65 and 69 years old.

**Table 1:** Baseline characteristics of the subjects of the study

	Control group	Probiotic group	*p* value
Age (years ± SD)	81.76 ± 7.2	83.79 ± 6.5	0.148
Male/female (%)	35.3/64.7	32/68	0.724
BMI (Kg/m^2^, mean ± SD)	26.3 ± 5.1	26.3 ± 3.5	0.974
Current smoker (%)	6.7	0	0.110
Previous smoking habits (%)	13.3	21.1	0.321
Years as smokers (mean ± SD)	38.1 ± 13.1	27.5 ± 11.7	0.142

The diets of the volunteers were recorded during the study period. No significant differences were noted in the diets of the volunteers among the study groups or between baseline and the end of the intervention (data not shown). Therefore, the significant effects observed for the outcomes cannot be attributed to the participants’ different dietary habits.

### Influenza vaccination immune response

The antibody titres against the three viruses included in the vaccine were evaluated at baseline and at 4 weeks and 8 weeks after influenza vaccination. Seroconversion was defined as the proportion of vaccinated individuals who achieved a haemagglutination inhibition titre > 1:40 for at least one of the virus subtypes included in the vaccine. No significant differences were observed between the measurements taken at weeks 6 and 10 (4 and 8 weeks after vaccination) (*p* = 0.519). The percentage of responders was higher in the probiotic group than in the control group (93.1 and 72.7%, respectively). The odds of seroconversion for at least one of the antigens of the vaccine was 4.94 times higher in the probiotic group than in the control group (*p* = 0.036) (Table 2). Similar effects were observed when the definition of seroconversion was based on an at least four-fold increase of the haemagglutination inhibition (HAI) titre against the influenza virus over baseline (Table 2). No interaction of age or sex was detected (*p* = 0.146 and *p* = 0.981, respectively).

**Table 2:** Seroconversion in subjects of the study

Seroconversion	Control group (% responders)	Probiotic group (% responders)	Increase probiotic vs control (%)	OR ajdusted	95% confidence interval (LL–UL)	*p* value
Seroconversion (titre > 1:40)	72.7	93.1	28.1	4.94	0.90–51.45	**0.036**
Seroconversion (increase > 4 titers)	75.0	92.1	22.8	3.05	1.33–7.65	**0.012**

Statistically significant values (*p* < 0.05) are given in bold

No significant differences were detected in the immunoglobulin A and G or the cytokine IL-4, IL-10 and TNF-alpha concentrations (Table 3).

**Table 3:** Immunological parameters

	Control group				Probiotic group			
	Baseline	2 weeks	6 weeks	10 weeks	Baseline	2 weeks	6 weeks	10 weeks
Ig A (mg/dL)	3.65 ± 2.4	3.67 ± 2.4	3.91 ± 2.2	3.81 ± 2.2	4.22 ± 2.5	3.93 ± 2.1	4.13 ± 2.3	4.24 ± 2.3
Ig G(mg/dL)	8.83 ± 6.9	9.16 ± 7.1	10.28 ± 7.6	9.91 ± 7.3	10.05 ± 7.9	10.42 ± 9.7	10.45 ± 8.1	11.08 ± 10.1
IL-4(pg/mL)	0.81 ± 0.5	0.79 ± 0.8	0.94 ± 0.5	0.83 ± 0.4	0.78 ± 0.5	0.80 ± 0.4	0.78 ± 0.4	71 ± 0.3
IL-10(pg/mL)	1.92 ± 2.7	1.81 ± 2.4	2.47 ± 3.6	2.56 ± 3.4	3.02 ± 5.3	3.65 ± 8.4	3.47 ± 9.1	2.67 ± 5.1
TNF-alpha (pg/mL)	3.61 ± 6.0	3.52 ± 5.3	4.93 ± 6.5	5.11 ± 8.5	7.98 ± 12.7	5.30 ± 8.7	7.07 ± 9.3	7.61 ± 9.5

Data are showed as mean of concentration ± SD

### Influenza-like illness and respiratory symptoms

The incidence of ILI was 46.3% lower in the probiotic group than in the control group, but the difference was not significant (*p* = 0.194). The incidences of symptoms usually associated with respiratory infections were lower in the probiotic group than in the control group, although the differences reached significance for only sore throat (Table 4). The incidence of local respiratory symptoms (sore throat, cough and/or nasal congestion) was approximately 48% lower in the probiotic group than in the control group (*p* = 0.007).

**Table 4:** Incidence of symptoms related to respiratory infections

	Symptoms usually associated to respiratory infections							Local symptoms^a^	ILI^b^
	Cough	Nasal congest	Sore throat	Headache	Muscle/bone pain	Fever	Tiredness		
Control IR(SD)	0.422 (0.70)	0.444 (0.92)	0.356 (0.57)	0.267 (0.75)	0.111 (0.32)	0.133 (0.34)	0.178 (0.39)	1.222 (1.98)	0.244 (0.43)
Probiotic IR (SD)	0.289 (0.57)	0.237 (0.49)	0.105 (0.39)	0.080 (0.27)	0.105 (0.39)	0.132 (0.34)	0.079 (0.27)	0.632 (1.15)	0.131 (0.34)
IR decrease (%)	31.5	46.6	70.5	70.0	5.4	0.75	55.6	48.3	46.3
*p* value	0.319	0.117	**0.029**	**0.059**	0.934	0.982	0.231	**0.007**	0.194

Statistically significant values (*p* < 0.05) are given in bold

^a^ Incidence of local respiratory symptoms (sore throat, cough and/or nasal congestion)

^b^*ILI* influenza-like illness

Logistic regression models were applied to obtain the adjusted estimates for the incidence ratios for each symptom in the seroconverted patients, but no significant interactions were found (*p* > 0.05).

The consumption of analgesic and antibiotic treatments was recorded during the study (Table 5). The administration of antibiotics was similar in both groups; however, the administration of analgesic treatments was 86% lower in the probiotic group (*p* = 0.008). A logistic model was applied to obtain the adjusted odds ratio (OR) for analgesic consumption by age and sex. No significant interactions of age or sex were detected (*p* = 0.316 and *p* = 0.929, respectively). The odds of analgesic consumption were significantly lower for the probiotic group (more than 6 times) than for the control group (OR = 0.151; 95% CI 0.022–0.641; *p* = 0.021).

**Table 5:** Pharmacological treatments related to symptoms of respiratory infections

	Antibiotic	Analgesic
Control IR (SD)	0.200 (0.40)	0.378 (0.81)
Probiotic IR (SD)	0.316 (0.57)	0.053 (0.23)
Incidence rate decrease (%)	− 58	86.0
*p* value	0.300	**0.008**

Statistically significant values (*p* < 0.05) are given in bold

### Gastrointestinal symptoms

The incidence of gastrointestinal symptoms (nausea/vomiting or lack of appetite) was also evaluated. The incidence of these symptoms was lower in the probiotic group than in the control group (0.105 ± 0.39 vs 0.267 ± 0.84); however, the difference was not significant (*p* = 0.651).

## Discussion

The elderly population is more susceptible to infectious diseases, owing to loss of the ability of the immune system to function [3, 4]. Vaccination is strongly recommended for this population because common infectious diseases, such as the flu, have a highly negative effect. However, the immune response defect provokes a lower level of protection after vaccination. Several strategies aimed at improving the response to vaccination in this population have been evaluated, including the use of probiotics. The present study demonstrates that administration of Lc *K8* significantly increases the percentage of responders to the vaccine. Despite the higher percentage of responders to the vaccine in the probiotic group, significant differences in the incidence of flu were not detected between groups. However, the diagnosis of flu was based on a confluence of symptoms rather than viral detection.

Interestingly, when the symptoms associated with respiratory infections were analysed, we observed a significant decrease in the incidence of symptoms, especially those related to upper respiratory infections. The effects of probiotics on upper respiratory infections have been previously reported for other strains. A Cochrane meta-analysis of 10 clinical trials involving a total of 3451 infants, children and adults has found that probiotics are more beneficial than placebo in terms of infection prevention [7]. Moreover, probiotics decrease the rate of acute upper respiratory tract infections and frequency of antibiotic use but also decrease of the duration of each single episode have been reported [7, 11].

Most studies include long intervention periods (months), and the incidence of respiratory infections is analysed during the intervention period [7, 22]. The coryniformis study is notable in that the intervention period was short (2 weeks), and the incidence of symptoms associated with respiratory infections was evaluated for 5 months after the probiotic intervention. Therefore, the effect of Lc K8 is not limited to the period of probiotic administration but is maintained over time. The permanence of the effect after treatment is discontinued which might have been due to the improved antibody response to the influenza-induced probiotic strain in response to influenza vaccination. However, most respiratory infections were not related to flu events and were identified as a common cold, which would involve another type of virus not included in the vaccine. Therefore, non-specific activation of the immune system might be involved in the protection against respiratory infections. Another hypothesis that might explain the permanence of the effect is intestinal colonization by the probiotic strain, which would allow the strain to continue activating the immune system of individuals over time. Nevertheless, colonization of the intestine by probiotic bacteria is considered to be transient. Thus, this colonization is not expected to be maintained for months after the cessation of ingestion, although some authors have detected permanent colonization at a very low level [23, 24].

In the Lc K8 study, no effects have been detected on the plasma cytokine and total immunoglobulin concentrations after the probiotic intervention. A previous study performed with the same probiotic strain in a hepatitis A vaccination protocol in a healthy adult population, has shown an adjuvant effect of the *L. coryniformis* strain, as evidenced by an increase in the production of specific antibodies against the antigens present in the hepatitis vaccine [18]. In the Redondo study, no effects attributed to Lc K8 administration have been observed on the cytokine concentration in the blood, and only a slight increase has been observed in the proportion of memory T lymphocytes (CD3 + CD4 + CD45RO+). As previously mentioned, because the effect of Lc K8 is observed mainly on local respiratory symptoms usually associated with the common cold, the effect might have been due to a general effect on the immune system instead of a specific higher response against the flu vaccine. In fact, a higher innate response has been related to the administration of probiotic strains. The polymorphonuclear (PMN) cell phagocytic capacity and natural killer (NK) cell activity have been identified as key immune functions against infection. Research in the elderly has shown that low NK activity is associated with the development of infectious diseases; thus, strategies to reinforce this activity should improve the immune response in this population [25, 26]. Several studies have shown the effect of probiotic strains on NK activity, although the results in the elderly vary and are strain and study dependent [10, 27, 28]. A previous study performed in healthy adults with a combination of Lc K8 and a *L. gasseri* (CECT5714) strain has found an increase in the proportion of NK cells and the phagocytic activity of monocytes and PMN cells [15]. However, the effects cannot be attributed solely to the *L. coryniformis* strain because a combination of strains was used.

Therefore, although the immune response of the elderly population was improved by increasing specific antibodies against the flu vaccine, the mediators of this immune effect should be clarified in future studies.

Interestingly, in the Lc K8 study, the consumption of analgesics was significantly lower (86%) in the elderly group who received the probiotic strain than in the control group. This decrease was consistent with the decrease in the incidence of symptoms observed in the probiotic group. The lower analgesic consumption is relevant from two perspectives. First, adverse effects, such as gastrointestinal problems, are usually associated with analgesic consumption [29, 30]. Moreover, analgesics may be counterproductive for elderly people because of possible interactions with concomitant pharmacological treatments or their special medical conditions [31]. Second, the decrease in analgesic treatments would affect health expenditures derived from the care of the elderly population.

In conclusion, the administration of *L. coryniformis* K8 CECT5711 to the elderly population increases the immune response against the influenza vaccine and decreases symptoms associated with respiratory infections. Probiotic administration may be a natural and safe strategy to improve the efficacy of vaccines and to protect against common respiratory infections in susceptible populations.
